# Singlet Exciton Lifetimes in Conjugated Polymer Films for Organic Solar Cells

**DOI:** 10.3390/polym8010014

**Published:** 2016-01-13

**Authors:** Stoichko D. Dimitrov, Bob C. Schroeder, Christian B. Nielsen, Hugo Bronstein, Zhuping Fei, Iain McCulloch, Martin Heeney, James R. Durrant

**Affiliations:** 1 Centre for Plastic Electronics, Department of Chemistry, Imperial College London, Exhibition Road, London SW7 2AZ, UK; c.nielsen@imperial.ac.uk (C.B.N.); z.fei@imperial.ac.uk (Z.F.); i.mcculloch@imperial.ac.uk (I.M.); m.heeney@imperial.ac.uk (M.H.); j.durrant@imperial.ac.uk (J.R.D.); 2 Department of Chemical Engineering, Stanford University, Stanford, CA 94305, USA; bschroeder@stanford.edu; 3 Department of Chemistry, University College London, London WC1H 0AJ, UK; h.bronstein@ucl.ac.uk; 4 SPERC, Physical Sciences and Engineering Division, King Abdullah University of Science and Technology (KAUST), Thuwal 23955–6900, Saudi Arabia

**Keywords:** excited states, diffusion, energy gap law, non-radiative, ultrafast transient absorption spectroscopy

## Abstract

The lifetime of singlet excitons in conjugated polymer films is a key factor taken into account during organic solar cell device optimization. It determines the singlet exciton diffusion lengths in polymer films and has a direct impact on the photocurrent generation by organic solar cell devices. However, very little is known about the material properties controlling the lifetimes of singlet excitons, with most of our knowledge originating from studies of small organic molecules. Herein, we provide a brief summary of the nature of the excited states in conjugated polymer films and then present an analysis of the singlet exciton lifetimes of 16 semiconducting polymers. The exciton lifetimes of seven of the studied polymers were measured using ultrafast transient absorption spectroscopy and compared to the lifetimes of seven of the most common photoactive polymers found in the literature. A plot of the logarithm of the rate of exciton decay *vs.* the polymer optical bandgap reveals a medium correlation between lifetime and bandgap, thus suggesting that the Energy Gap Law may be valid for these systems. This therefore suggests that small bandgap polymers can suffer from short exciton lifetimes, which may limit their performance in organic solar cell devices. In addition, the impact of film crystallinity on the exciton lifetime was assessed for a small bandgap diketopyrrolopyrrole co-polymer. It is observed that the increase of polymer film crystallinity leads to reduction in exciton lifetime and optical bandgap again in agreement with the Energy Gap Law.

## 1. Introduction

Organic solar cells (OSC) have power conversion efficiencies that are now surpassing 11% which is a five-fold increase from the efficiency of the first reported OSC device 30 years ago [1,2,3]. However, significant further performance improvements are required for the successful translation of this technology into a commercial product. Conjugated polymers are the most widely used light harvesting materials for OSC. In the device active layer, they are normally blended with a soluble derivative of the fullerene C60 or C70 or with a high electron affinity aromatic molecule to create an electron donor-acceptor (D-A) pair that has an energy landscape that favors photocurrent generation. Unlike most inorganic semiconductors, the absorption of light by conjugated polymers does not lead to the direct generation of free charges, but instead coulombically-bound pairs of electrons and holes are created [4]. These so called excitons have relatively short lifetimes and limited diffusivities [5,6,7]. They have binding energies that are an order of magnitude bigger than the thermal energy (kT) and their dissociation requires an external force which in the OSC devices is provided by the molecular orbital energy offset at the D-A interface [8].

Polymer singlet excitons are the precursors for charge photogeneration taking place at the D-A interface and despite of their importance for OSC performance, relatively little is known about their properties and how we can control them. In particular, there are not clear strategies for the optimization of the exciton lifetime in conjugated polymer films. Therefore, the aim of this work is to provide an analysis of the singlet exciton lifetimes of 16 different conjugated polymers and to test the validity of the Energy Gap Law in thin polymer films [9,10]. The paper starts with a brief overview of the nature of the excited states in conjugated polymer films covering exciton diffusion and lifetimes. Then, the results from transient absorption spectroscopy measurements of the exciton lifetimes of seven polymers are presented alongside the lifetimes of seven popular polymers widely used for OSC fabrication. Then, three films of the co-polymer BTT-DPP (Poly[(5-decylbenzo[1,2-b:3,4-b′:5,6-d″]trithiophene-2,8-diyl)-*alt*-*co*-(3,6-bis(2-thienyl)-2,5-dihydro-2,5-di(2-octyldodecyl)pyrrolo[3,4c]pyrrolo-1,4-dione-5,5′-diyl)]) with different molecular weights were characterized to assess the impact of film crystallinity on the exciton lifetime.

### 1.1. Excited States in Conjugated Polymers

The excited states of conjugated polymers are coulombically-bound electron-hole pairs that resemble molecular excited states [4]. They are called singlets when their spin is 0 and triplets when their spin is 1. The singlets and triplets vary broadly in properties but only singlets are populated upon polymer light absorption. This is because of the spin forbidden nature of the transition from a singlet ground state to a triplet excited state. Triplets are generated indirectly through intersystem crossing from a singlet exciton or through electron-hole recombination involving a spin flip [11,12,13,14]. Singlet and triplet excitons are both formed in OSC during device function but predominantly singlets are involved in charge photogeneration, while triplets are normally associated with undesirable recombination processes [11,12,13,14].

**Figure 1:**
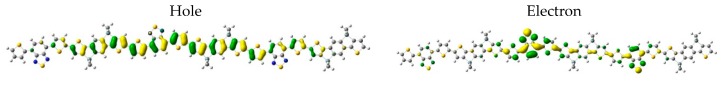
Charge density isosurface of the electron and hole natural transition orbitals of the lowest energy electronically excited state in the trimer of SiIDT-DTBT, depicting the charge density distribution upon excitation by light. Calculations were performed by TD-DFT B3LYP/6-31g* and are taken with permission from reference [13].

Singlet excitons can be broadly classified as intrachain and interchain [15,16,17]. In the former, the excited state extends over a single polymer chain and its delocalization is mainly limited by conformational disorder and chemical defects [18]. This is typically the case for homologous polymers like P3HT (Poly(3-hexylthiophene-2,5-diyl)). In push-pull type co-polymers the excited state is not evenly distributed along the polymer chain, [19] because the co-polymers consist of at least two different monomer units that have differing electron affinities. Therefore, the absorption of light by such co-polymers often results in intrachain partial charge separation that is driven by the difference in electron affinity between the monomers [20,21,22,23]. For example, light absorption by the co-polymer SiIDT-DTBT induces partial electron density localization on the benzothiadiazole (BT) unit, due to its stronger electron withdrawing character, while the hole is more uniformly delocalized along the whole polymer chain (Figure 1) [13]. Such push-pull polymers have been widely developed over the past ten years and are the main reason behind the significant improvements in organic solar cell device efficiencies, as they provide smaller optical bandgaps for better light harvesting and improved control over device photovoltages [2,21,24,25].

In addition to intrachain excited states, many conjugated polymers including co-polymers form interchain excited states that can be thought of as the excitons generated in molecular crystals, where the excitation can be shared between neighboring chromophores [26]. These occur frequently in molecular crystals and crystalline polymer films as a result of strong electronic interactions between the neighboring chromphores [27]. The exciton is a strongly bound electron-hole particle that has no net dipole moment and distinctive optical properties, such as well-defined vibronic peaks and strong 0-0 transitions. These optical signatures allow an easy identification of a high structural order in thin polymer films [28].

Another type of interchain excited state that has been observed in polymer films is the excimer that is an excited state complex of two stacked chromophores formed dynamically through molecular rearrangements after light absorption [29]. The excimer cannot be directly excited through light absorption and is identified by its distinctively broad and featureless emission spectrum that is red-shifted from the monomer emission [30]. Excimers are frequently observed in solutions of aromatic molecules, where solvent and molecular geometrical rearrangements are possible. In thin polymer films, however, the movement of the chromophores is highly restricted due to steric hindrance caused by dense polymer packing. Despite of this, excimer formation still takes place in films and on surprisingly fast timescales (picoseconds), possibly because of the existence of pre-associated complexes that require only a small geometrical reorganization [4]. It has been demonstrated that the yield of excimers in polymer films can be significant reaching up to 30% for highly crystalline P3HT domains [31,32]. Rumbles *et al.* have also pointed out that in addition to excimers and excitons, polymer films generate a low yield of long-lived polaron states [4,33]. These are electrons and holes that are coupled to the molecular vibrations and are able to diffuse through the film [26]. Due to their low yields, the polarons will not be further discussed in this article. We note that excimers and excitons are often difficult to distinguish spectroscopically. Therefore, in the studies of excited state diffusion in polymer films these are generally referred to as singlet excitons.

### 1.2. Exciton Diffusion in Polymer Films

Singlet exciton diffusion is one of the key material parameters that determine OSC device performance. It can be described as the migration of energy through a film via series of energy transfer steps. In its simplest form, each step can be understood as a dipole-dipole induced non-radiative energy transfer process between the neighboring chromophores in the film that is best described by the Forster Resonance Energy Transfer mechanism [6,7,34]. In the OSC literature, the most widely cited figure of merit for exciton diffusion is the exciton diffusion length (*L_D_*) that is defined as the root mean square of the spatial displacement of the exciton from its origin during its lifetime. It, therefore, depends directly on the lifetime of the excitons as shown in the following expression:

(1) $$L_{D}=\sqrt{2ZD\tau }$$

where *Z* is equal to 1, 2, or 3 for the number of dimensions the exciton can travel, *D* is the diffusivity of the film expressed with units cm^2^·s^−1^ and τ is the exciton lifetime.

The singlet exciton diffusion lengths of many semiconducting polymers have been reported to be between 5 and 10 nm; these are approximately an order of magnitude smaller than the light absorption depth of a typical polymer film [5,6,7,35,36]. As a result, the majority of OSC devices developed to date have a photoactive layer that is a fine blend of a polymer and fullerene rather than a bi-layer. This device structure allows for relatively efficient light harvesting and exciton dissociation to be achieved [37]. However, the intimate mixing of the polymer and fullerene creates a very high density of the polymer-fullerene interface that leads to an increased probability for electron-hole recombination and hence reduced device photovoltages and fill factors. One way to avoid this issue is to optimize the microstructure of the blend by creating well defined polymer and fullerene interpenetrating networks for excellent charge transport properties [38,39], as recently demonstrated by Yan *et al.* with the best performing single-junction polymer:fullerene solar cells reported to date [2]. However, a high degree of film order often coincides with the formation of large polymer domains which may limit the yield of exciton dissociation and hence photocurrent generation [40]. Therefore, the development of polymers with longer exciton lifetimes and longer exciton diffusion lengths can provide an additional degree of freedom for microstructural film optimization for more efficient charge transport and higher power conversion efficiencies.

### 1.3. Exciton Lifetime

As shown in Equation (1), the exciton lifetime is one of two parameters that determine the polymer exciton diffusion lengths. Longer exciton lifetimes should in principle translate into longer diffusion lengths. Nevertheless, our ability to control the polymer exciton lifetime, either synthetically or via material processing is currently very limited. The lifetime of singlet excitons is determined by both radiative and non-radiative photophysical processes and can be expressed with the following equation [41]:

(2) τ_AVE_ = 1/(*k*_R_ + *k*_NR_)

where τ_AVE_ is the measured lifetime, *k*_R_ is the rate of radiative decay, and *k*_NR_ is the average rate of non-radiative recombination decay. The exciton lifetime is also directly related to the fluorescence quantum yield (QY) via the equation:

(3) QY = *k*_R_/(*k*_R_ + *k*_NR_)

In the case of large hydrocarbon molecules τ_AVE_ is normally dominated by the rate of non-radiative recombination decay. As a result, large molecular complexes and polymers usually have very low QY. For example, the QY of the thin films of the benchmark polymers P3HT, PTB7, and MEH-PPV are <2% [42,43,44], although there are some exceptions to this empirical observation, as reported for PFO (QY of 53%) [45]. The dominance of the non-radiative photophysical processes is further enhanced in thin films, which is evident in experiments comparing the exciton lifetime and QY of polymers in solution and films. Therefore, for the rest of this manuscript we will focus on examining the non-radiative decay of singlet excitons to ground state in conjugated polymer films.

Non-radiative electronic transitions in molecules and polymers include internal conversion, intersystem crossing, electron transfer, and electron-hole recombination. It is generally known that the rate constant of these processes depends on the energy gap between the initial and final states involved [46,47], which is well demonstrated in the Marcus Theory of non-adiabatic electron transfer and in the Energy Gap Law [9,10]. The latter describes the rate of unimolecular non-radiative decay between two weakly coupled electronic states, such as the lowest energy singlet excited state (S_1_) and the singlet ground state (S_0_). Therefore, the Energy Gap Law can be directly related to the rate of exciton decay to S_0_ (inversely proportional to the exciton lifetime) and it can be most simply presented as:

(4) *k*_NR_ α e^−γΔ*E*^

where γ is a molecular parameter that includes the highest energy vibrational mode involved in the non-radiative transition and Δ*E* is the energy difference between the potential minima of S_1_ and S_0_.

The Energy Gap Law has been successfully applied to describe the non-radiative transitions in variety of systems including aromatic molecules and heavy atom complexes, as well as the triplet exciton decay in conjugated polymer films [47,48,49,50,51]. Nevertheless, the validity of this law has not been tested for the rate of exciton decay from S_1_ to S_0_ in conjugated polymer films. We, therefore, collected the singlet exciton lifetimes for fourteen conjugated polymers that have been employed for OSC fabrication to study the relationship between polymer bandgap and exciton lifetime, if any. The structures of the polymers are presented in the SI and include some of the most widely studied polymers to date.

## 2. Materials and Methods

The materials studied herein have been synthesized via established synthetic procedures and their optical properties characterized. The full names of the polymers are as follows: 

BTT-DPP [52]: Poly[(5-decylbenzo[1,2-b:3,4-b′:5,6-d″]trithiophene-2,8-diyl)-*alt*-*co*-(3,6-bis(2-thienyl)-2,5-dihydro-2,5-di(2-octyldodecyl)pyrrolo[3,4c]pyrrolo-1,4-dione-5,5′-diyl)];

DPP-TT-T [21]: Poly[[2,5-bis(2-octyldodecyl)-2,3,5,6-tetrahydro-3,6-dioxopyrrolo[3,4-c]pyrrole-1,4-diyl]-*alt*-[[2,2′-(2,5-thiophene)bis-thieno[3,2-b]thiophen]-5,5′-diyl]];

SiIDT-BT [53]: Poly[(2,1,3-benzothiadiazole-4,7-diyl)-*alt*-(4,9-dihydro-4,4,9,9-tetraoctylbenzo [1″,2″:4,5;4″,5″:4’,5’]bissilolo[3,2-b:3’,2’-b’]dithiophene-2,7-diyl)];

SiIDT-2FBT [54]: Poly[(5,6-difluoro-2,1,3-benzothiadiazole-4,7-diyl)-*alt*-(4,9-dihydro-4,4,9,9-tetraoctylbenzo[1″,2″:4,5;4″,5″:4’,5’]bissilolo[3,2-b:3’,2’-b’]dithiophene-2,7-diyl)];

Bu-GeDT [55]: Poly(3,5-didodecyl)-4,4′-di-*n*-butyldithieno[3,2-b:2′,3′-d]-germole)-2,6-diyl-*alt*-(2,2′-bithiophene)-5,5′-diyl);

Bu-SiDT [55]: Poly(3,5-didodecyl)-4,4′-di-*n*-butyldithieno[3,2-b:2′,3′-d]silole)-2,6-diyl-*alt*-(2,2′-bithiophene)-5,5′-diyl);

SiIDT-TPD synthesis is provided in the SI [53]: Poly[(5,6-dihydro-5-octyl-4,6-dioxo-4H-thieno[3,4-c]pyrrole-1,3-diyl)-*alt*-(4,9-dihydro-4,4,9,9-tetraoctylbenzo[1″,2″:4,5;4″,5″:4’,5’]bissilolo[3,2-b:3’,2’-b’]dithiophene-2,7-diyl)];

TTP [56]: poly(4,4’-didecyl-2,2’-bithiophene-co-phenyl);

PCDTBT: poly[*N*-9’-heptadecanyl-2,7-carbazole-*alt*-5,5-(4’,7’-di-2-thienyl-2’,1’,3’-benzothiadiazole)];

PCPDTBT: Poly[2,6-(4,4-bis-(2-ethylhexyl)-4H-cyclopenta [2,1-b;3,4-b′]dithiophene)-*alt*-4,7(2,1,3-benzothiadiazole)];

PFO: Poly(9,9-di-n-octylfluorenyl-2,7-diyl);

PTB7: Poly({4,8-bis[(2-ethylhexyl)oxy]benzo[1,2-b:4,5-b′]dithiophene-2,6-diyl}{3-fluoro-2-[(2-ethylhexyl)carbonyl]thieno[3,4-b]thiophenediyl});

MEH-PPV: Poly[2-methoxy-5-(2-ethylhexyloxy)-1,4-phenylenevinylene].

The thin polymer films were fabricated via spin-coating from chlorobenzene or di-chlorobenzene solutions on glass substrates. The absorption spectra of the films were measured with a PerkinElmer Lambda 25 spectrometer in air. The optical bandgaps of the films were estimated from the onset of the lowest absorption peak.

Femtosecond transient absorption spectroscopy was carried out using a commercially available transient absorption spectrometer, HELIOS (Ultrafast systems, Sarasota, FL, USA). Samples were excited with light pulses with a wavelength matching the lowest energy absorption maxima of the polymers. The excitation was generated by an optical parametric amplifier, TOPAS (Light conversion, Vilnius, Lithuania) seeded with the output of an 800 nm, ~100 fs, 1kHz Solstice Ti:sapphire regenerative amplifier (Spectra-Physics, Newport Ltd. , Santa Clara, CA, USA). The probe pulses were generated in a sapphire plate and detected with an InGaAs CMOS camera.

## 3. Results and Discussion

Table 1 presents the abbreviations of the polymer names, their optical bandgaps and exciton lifetimes as measured in thin films. The exciton lifetimes of the first seven polymer films were estimated using high sensitivity ultrafast transient absorption spectroscopy. In all measurements, the light excitation densities were kept sufficiently low to avoid a non-linear response from the samples. The exciton lifetimes were received by exponential fitting of the singlet exciton absorption decays presented in the SI and extracted from the NIR transient absorption spectra of the polymers (included in the Supplementary Materials). In the case of multi-exponential decays, the average exciton lifetimes were estimated by calculating the weighted average of all time constants received from the fits. In order to expand the number of data points and compare our results to previously published values, the lifetimes of seven additional polymers were taken from the literature [42,44,45,57,58,59,60,61,62,63,64,65,66,67,68,69]. These include some of the most widely studied polymers to date: P3HT, PCDTBT, PCPDTBT, PFO, PTB7, and MEH-PPV (full polymer names included in the Materials and Methods Section). It is worth noting that polymer exciton lifetimes published in literature vary widely, probably because of differences in the source of the polymers and their film processing conditions. Another possible source of error is the difference in the spectroscopic techniques used and the experimental conditions. This variation in lifetimes is included as error bars in the current analysis of the exciton lifetimes.

**Table 1:** Optical bandgaps and exciton lifetimes of the polymers analyzed in this study. The full names of the polymers are included in the Materials and Methods section.

Polymer	Optical bandgap (eV)	Exciton lifetime (ps)
BTT-DPP	1.33	18 ± 0.9 ^a^
DPP-TT-T	1.38	36.8 ± 1.5 ^a^
SiIDT-BT	1.80	112 ± 4 ^a^
SiIDT-2FBT	1.80	175 ± 7 ^a^
APFO-3	1.93	400 ± 83 ^a^
SiIDT-TPD	2.00	70 ± 6 ^a^
TTP	2.60	127 ± 5 ^a^
PCPDTBT	1.43	78 ^b^
PTB7	1.65	93 ± 48 ^b^
PCDTBT	1.86	463 ± 193 ^b^
PBTTT	1.90	175 ^b^
P3HT	1.95	422 ± 150 ^b^
MEH-PPV	2.11	210 ± 79 ^b^
PFO	2.80	430 ^b^

^a^ Lifetimes measured using transient absorption spectroscopy; ^b^ Average lifetimes from published work, measured via ether transient absorption or fluorescence spectroscopy [42,44,45,57,58,59,60,61,62,63,64,65,66,67,68,69].

**Figure 2:**
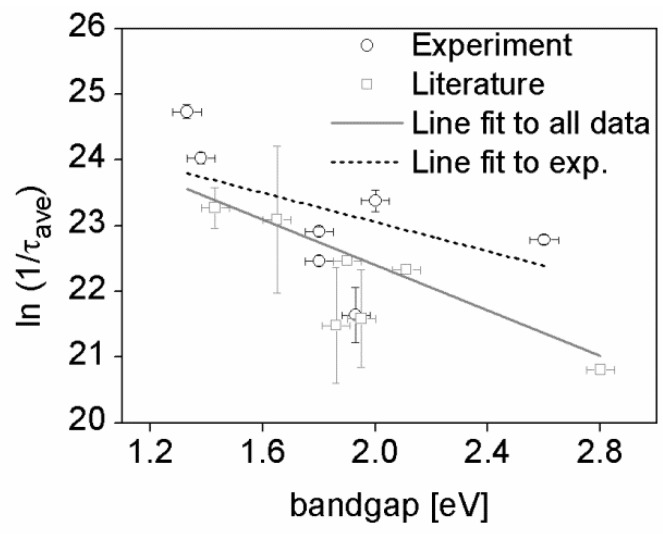
The natural logarithm of the rate of singlet exciton decay plotted as a function of optical bandgap for 16 conjugated polymer films included in Table 1. The open circles represent the experimentally determined rates of exciton decay of the polymer films studied, estimated using ultrafast transient absorption spectroscopy. The error bars represent the square root of the mean residual variance of the exciton lifetime as received from the exponential fits of the exciton decays. The open squares represent the rates of exciton decay for popular polymers as extracted from the literature. The error bars represent the standard deviation of the exciton lifetimes found in the literature for each polymer. The black line is the best fit straight line to all data points showing a negative slope and the broken line is the best fit straight line to the seven polymers characterized herein.

Figure 2 presents a plot of the natural logarithm of the inverse of the exciton lifetime *vs.* the optical bandgap of all thin polymer films presented in Table 1, which allows us to test the validity of the Energy Gap Law for the S_1_ to S_0_ transition in the polymers examined here. The exciton lifetime of PFO is corrected using Equation (2) for the high QY of this polymer (53%); it, therefore, represents the non-radiative recombination decay of the PFO singlet exciton. A similar correction was carried out for PCPDTBT (QY of 6%) [63,67], although the received non-radiative decay constant is well within the standard deviation of the lifetimes measured for this polymer. The scatter data in Figure 2 was fitted to a line function which yielded a low adjusted root square value of 0.40, which reflects the high variation in exciton lifetimes. A relatively high Pearson’s correlation of −0.66 is received from the fit, which allows us to suggest that there is a correlation between the optical bandgap and the rate of exciton decay of the polymers that can be assigned to the Energy Gap Law. In addition, we attempted to fit only the seven polymers measured in this study, separately from the data points taken from the literature. The fit is presented in Figure 2 as a black broken line and it shows a very low adjusted root square value of 0.30 and a relatively high Pearson’s correlation of −0.65. From both fits we find a negative correlation between the rate of decay and the optical bandgap, which means that smaller bandgap polymers should be expected to have shorter exciton lifetimes. However, it is very clear that there are other factors that control the singlet exciton lifetimes of the polymers, thus suggesting that further work is required to allow us to confirm undoubtedly the validity of the Energy Gap Law. For example, the NIR spectra of the polymer APFO-3 (shown in the Supplementary Materials) exhibit two excited state absorption peaks, which indicate that two excited state species with different energetics are generated in this polymer. Therefore, a poor correlation between optical bandgap and exciton lifetime can be expected, which is indeed observed for APFO-3 that is also the biggest outlier point from our fit to the experimentally-determined data in Figure 2.

According to the Energy Gap Law, the slope of the linear fit (γ) depends on the geometrical rearrangements experienced by the polymers during their non-radiative decay from the excited to the ground state. It also includes the maximum energy vibration involved in the decay. Considering the relatively low confidence in the fit in Figure 2, a low value of γ (−1.7 ± 0.5 eV^−1^) is extracted [48]; thus, suggesting that for the materials studied the singlet exciton decay to ground state is likely to involve a high distortion of the polymer equilibrium geometries. This result is not surprising based on the relatively high Stokes shifts observed in conjugated polymers due to their high disorder. It also provides a possible reasoning for the large variation in exciton lifetimes seen here, possibly originating from differences in film crystallinity, although no obvious trends between film crystallinity and exciton lifetime can be observed in Figure 2.

**Figure 3:**
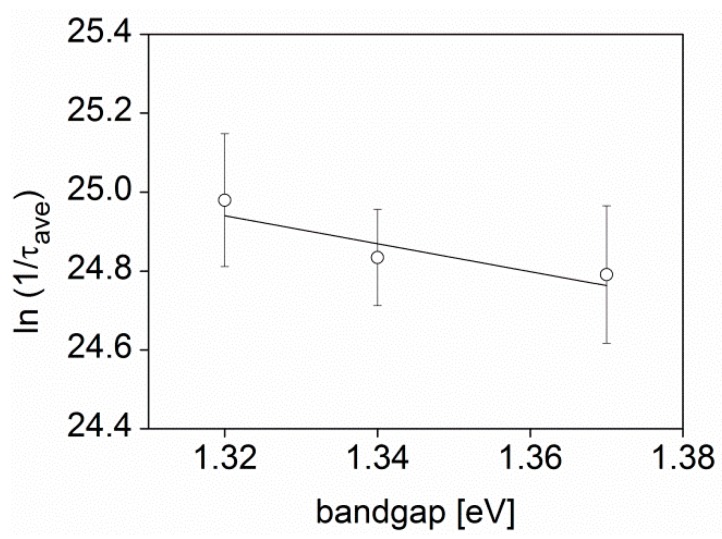
The natural logarithm of the rate of singlet exciton decay plotted as a function of optical bandgap for three BTT-DPP polymers with different molecular weight and film crystallinity. The open circles represent the experimentally determined rates of exciton decay of polymer films studied herein, estimated using ultrafast transient absorption spectroscopy. The error bars represent the square root of the mean residual variance of the exciton lifetime as received from the exponential fits of the exciton decays. The black line is the best fit straight line to all data points showing a negative slope with a value of −3.5 ± 1.9 eV^−1^.

To test the importance of film crystallinity on the exciton lifetime, three BTT-DPP polymers with differing molecular weights were studied using transient absorption spectroscopy [52]. Previously published WAXS measurements have indicated that the crystallinity of the BTT-DPP films increases with decreasing molecular weight of the polymer: lower molecular weight polymers produce more crystalline films [70]. According to Table 2, the lifetime of the singlet excitons in the three BTT-DPP films also decreases with increasing molecular weight. Furthermore, a careful examination of the absorption spectra of the BTT-DPP films (see Supplementary Materials) reveals that the changes in molecular weight impact not only the exciton lifetime but also the optical bandgap of the polymers. This result is supported by recent reports showing that the morphological changes of organic films impact their molecular orbital energetics [71]. In Figure 3, we include an Energy Gap Law plot of the natural logarithm of the inverse of the exciton lifetime of the BTT-DPP films as a function of optical bandgap. The data was successfully fitted with a linear function producing a high confidence fit with Pearson’s correlation of −0.88 and medium adjusted root square value of 0.54. The fit also estimated a gradient of −3.5 ± 1.9 eV^−1^ which is in a good agreement with previously published values for molecules and polymers [48]. This indicates relatively small displacement of molecular geometry between the excited and ground states for this polymer. On the basis of these results, it is possible to conclude that the changes in BTT-DPP film crystallinity cause a change in both polymer exciton lifetime and optical bandgap that follow the Energy Gap Law.

**Table 2:** Optical bandgaps and exciton lifetimes of BTT-DPP polymer films with differing number average molecular weights.

Polymer name	Optical bandgap (eV)	Exciton lifetime (ps)
BTT-DPP 90 kg·mol^−1^	1.37	17.1 ± 1.5
BTT-DPP 73 kg·mol^−1^	1.34	16.4 ± 1.0
BTT-DPP 22 kg·mol^−1^	1.32	14.2 ± 1.2

## 4. Conclusions

The results presented herein have a particular significance for the development of materials for OSC, especially in lights of the extensive number of small bandgap materials with absorption up to and beyond 900 nanometres reported over the past five years. They suggest that smaller bandgap polymers should generally exhibit shorter exciton lifetimes than higher bandgap polymers because of an increase in their rate of non-radiative exciton decay that follows the Energy Gap Law. This reveals a fundamental limitation of small bandgap polymers for their use in OSC devices as light harvesting materials. Indeed, recent reports of small bandgap materials have shown that incomplete exciton dissociation is a key limiting factor for their device photocurrent generation properties [70,72].

Our analyses of the exciton lifetimes generate high variance plots with medium confidence fits which suggest that in addition to the bandgap there are other material parameters that control the exciton lifetimes. There are multiple possibilities, of which the most obvious ones are the diversity of chemical structures between the polymers used, the presence of different heteroatoms in their structures and the differences in film order/crystallinity. These factors may lead to the population of very different in nature excited states that may result in different exciton lifetimes, as discussed in Section 1.1. For example, our comparison of the exciton lifetime of the polymer BTT-DPP as a function of molecular weight shows clearly that film crystallinity impacts the exciton lifetime of the polymer and it can therefore be used as a general tool to increase or decrease the exciton lifetime in polymer films.

## Supplementary Materials

The following are available online at www.mdpi.com/2073-4360/8/1/14/s1, the synthesis of SiIDT-TPD; Table S1 includes the structures of the polymers and their transient absorption spectra and decays of; Figure S1 presents the steady state absorbance of the fractionated BTT-DPP polymer; and Table S2 presents the transient absorption spectra and decays of fractionated BTT-DPP.
