# Supplementary material for: Singlet Exciton Lifetimes in Conjugated Polymer Films for Organic Solar Cells
Source: Polymers (Basel). 2016 Jan 13;8(1):14. doi: 10.3390/polym8010014 (PMC6432597; doi:10.3390/polym8010014)
Supplement: Supplementary file 1 [file polymers-08-00014-s001.pdf]

Supplementary Materials: Singlet Exciton Lifetimes in Conjugated Polymer Films for Organic Solar Cells

Stoichko D. Dimitrov, Bob C. Schroeder, Christian B Nielsen, Hugo Bronstein, Zhuping Fei, Iain McCulloch, Martin Heeney and James R. Durrant

Table S1. Transient decays of the excited state absorption of the singlet excitons of the polymers studied. Multiple single wavelength decays are plotted to improve the quality of the data fitting and avoid complications from spectral inhomogeneity. The experimental data is included as scatter points. The solid lines represent exponential fits to the data. The kinetics of BTT-DPP, DPP-TT-T, SiIDT-2FBT, SiIDT-BT were well fitted to a single exponential function. The kinetics of SiIDT-TPD, TTP and APFO-3 were well fitted to a bi-exponential function. Exciton lifetimes presented in the main text were calculated from the weighted average of the lifetimes received from the fits. Due to more complex spectral decay dynamics, the lifetime of the APFO-3 exciton decay was taken from the second time constant received from the bi-exponential fit. Transient absorption spectra of the films are presented in the graph. The positive signals are assigned to exciton absorption while negative signal correspond to ground state bleaching. Both the bleaching and exciton absorption signals follow the same decay dynamics.

<table>
<tr><th>Polymer</th><th>Exciton decay</th><th>Transient absorption spectra</th></tr>
<tr><td>BTT-DPP
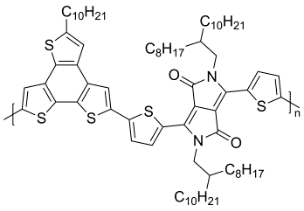</td><td>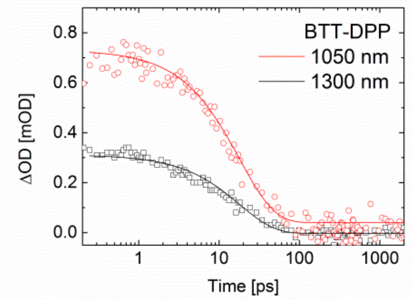</td><td>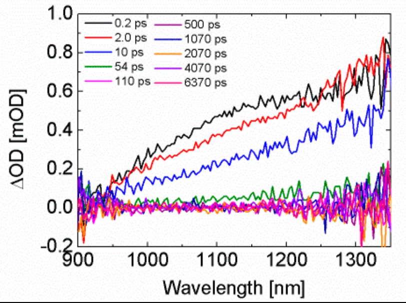</td></tr>
<tr><td>DPP-TT-T
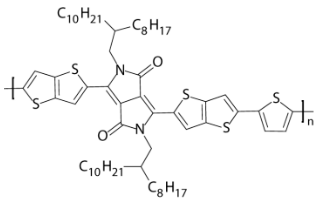</td><td>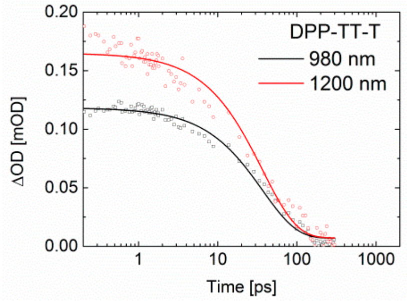</td><td>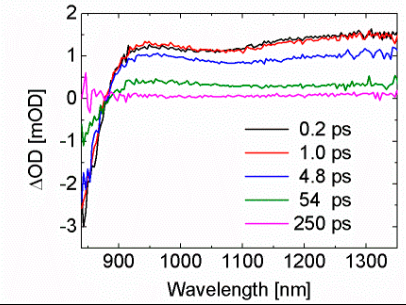</td></tr>
<tr><td>SiIDT-BT
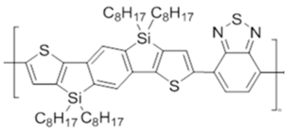</td><td>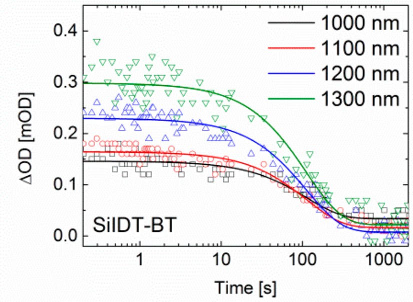</td><td>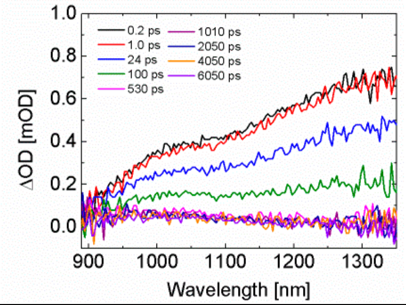</td></tr>
</table>

Table S1. *Cont.*

<table>
<tr><th>Polymer</th><th>Exciton decay</th><th>Transient absorption spectra</th></tr>
<tr><td>SiIDT-2FBT
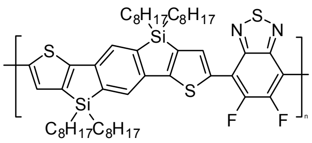</td><td>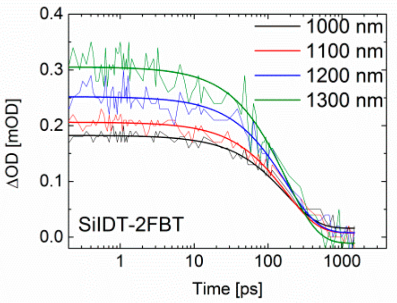</td><td>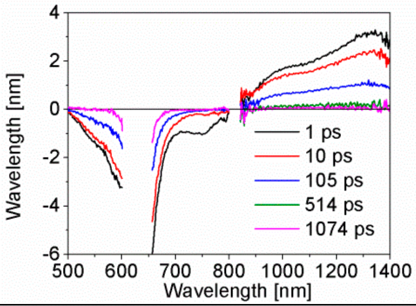</td></tr>
<tr><td>APFO-3
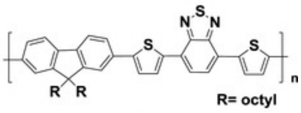</td><td>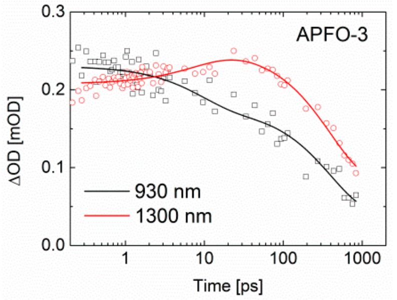</td><td>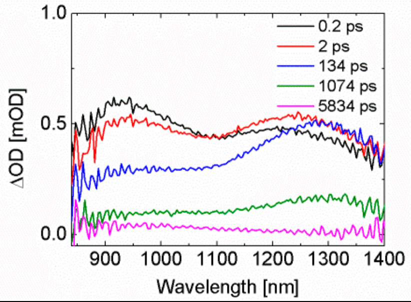</td></tr>
<tr><td>SiIDT-TPD
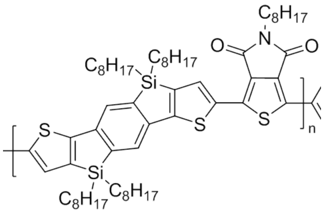</td><td>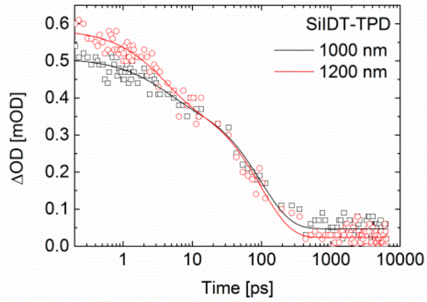</td><td>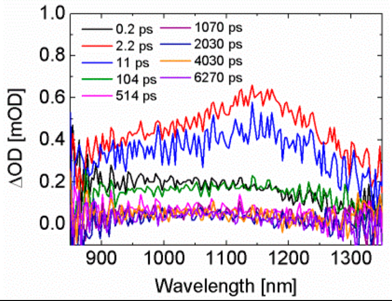</td></tr>
<tr><td>TTP
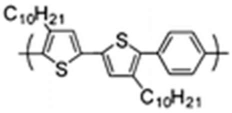</td><td>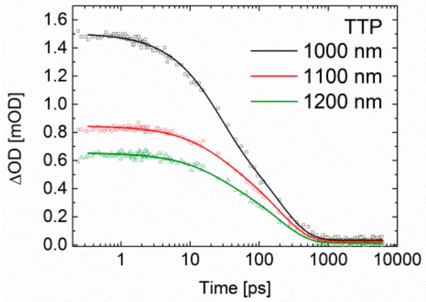</td><td>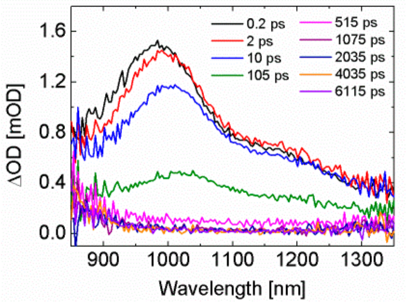</td></tr>
</table>

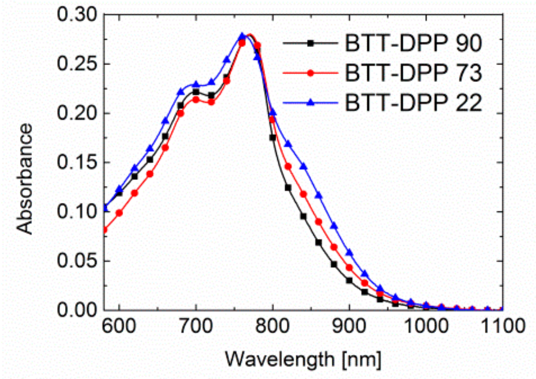

Figure S1. Steady-state absorbance spectra of fractionated BTT-DPP. Polymers with three different number average molecular weights were studied: 22, 73 and 90 kg.mol^{-1}.

Table S2. Exciton decays of the BTT-DPP polymers with different molecular weights. The exciton decays were fitted as in Table 1. The transient absorption spectra of the films are also presented depicting that exciton absorption (positive signal) and ground state bleaching (negative signal) follow the same decay dynamics.

<table>
<tr><th>Polymer</th><th>Exciton decay</th><th>Transient absorption spectra</th></tr>
<tr><td>BTT-DPP
90 kg mol-1</td><td>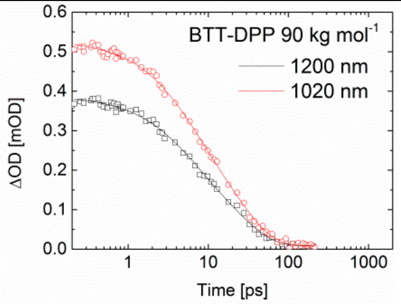
</td><td>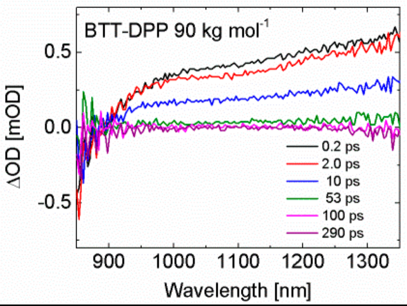
</td></tr>
<tr><td>BTT-DPP
73 kg mol-1</td><td>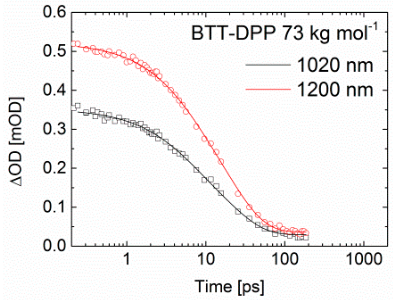
</td><td>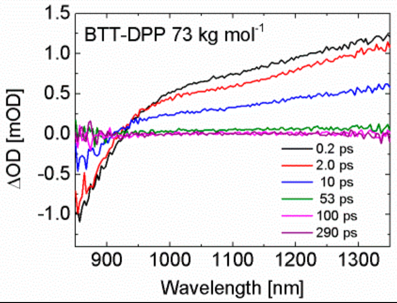
</td></tr>
<tr><td>BTT-DPP
22 kg mol-1</td><td>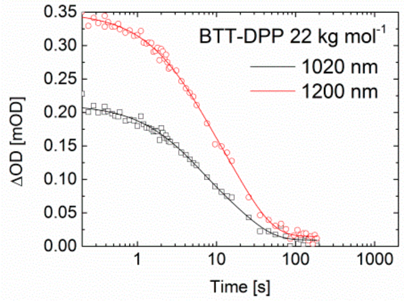
</td><td>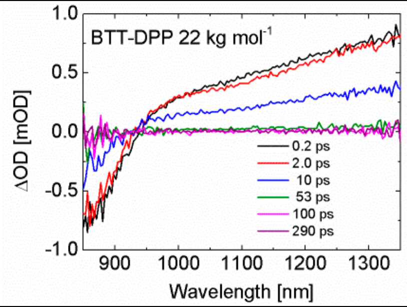
</td></tr>
</table>

Synthesis of SiIDT-TPD

A 5 mL microwave vial was charged with with 4,9-dihydro-4,4,9,9-tetraoctyl-2,7-bis(trimethylstannyl)-benzo[1′′,2′′:4,5;4′′,5′′:4′,5′]bissilolo[3,2-b:3′,2′-b′]dithiophene 1 (200 mg, 0.19 mmol), 1,3-dibromo-5-octyl-4H-thieno[3,4-c]pyrrole-4,6(5H)-dione (79 mg, 0.19 mmol), tris(dibenzylideneacetone)dipalladium (0) (3.4 mg, 3.7 µmol) and tri(o-tolyl) phosphine (4,5 mg, 15 µmol). The vial was sealed and anhydrous chlorobenzene (1 mL) was added. The obtained solution was degassed with argon for 30 min before The vial was subjected to microwave heating: 2 min at 100 °C, 2 min at 120 °C, 5 min at 140 °C, 5 min at 160 °C and 40 min at 170 °C. The polymer was end-capped by addition of 0.1 eq. of bromobenzene (2.0 µL, 19 µmol) before the reaction mixture was resubmitted to the microwave reactor, 1 minute at 100 °C, 1 minute at 120 °C, 2 min at 140 °C and 5 min at 160 °C. The polymeric solution was cooled down and 0.1 eq. of trimethyl(phenyl) stannane (3.4 µL, 19 µmol) was added by syringe. The reaction vial was subjected to the aforementioned temperature scheme to finalize the end-capping. After reaction, the crude polymer was precipitated in methanol, followed by Soxhlet extractions with acetone, hexane and chloroform during 24 h each. Remaining palladium residues were removed by treating a polymeric chloroform solution with an aqueous sodium diethyldithiocarbamate solution for 2 h at 50 °C under vigorous stirring. Afterwards the organic phase was separated from the aqueous phase and washed several times with water. The polymeric solution was concentrated under reduced pressure and precipitated into cold methanol. The polymer was filtered off and dried under high vacuum (142 mg, 0.16 mmol). M_n=17 kg·mol^{-1},

M_w=35 kg·mol^{-1}, $Đ_w$=2.05. 1H NMR (*o*-DCB-d4, 400 MHz, 40 °C): δ 8.44 (s, 2H), 7.99 (s, 2H), 3.96–3.53 (bm, 2H), 1.82–1.65 (bm, 2H), 1.64–1.48 (m, 8H), 1.38–1.02 (bm, 58H), 0.94–0.81 (m, 15H).

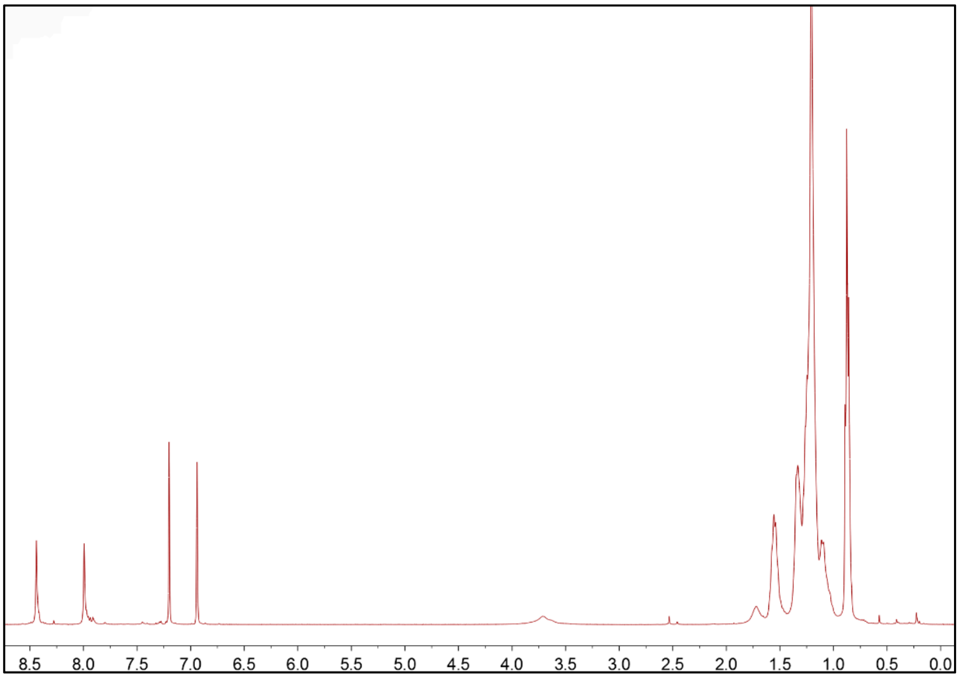

Figure S2. ^{1}H NMR spectrum of SiIDT-TPD.

© 2016 by the authors; licensee MDPI, Basel, Switzerland. This article is an open access article distributed under the terms and conditions of the Creative Commons by Attribution (CC-BY) license (http://creativecommons.org/licenses/by/4.0/).
